# Identification of rare and common variants in *BNIP3L*: a schizophrenia susceptibility gene

**DOI:** 10.1186/s40246-020-00266-4

**Published:** 2020-05-11

**Authors:** Juan Zhou, Chuanchuan Ma, Ke Wang, Xiuli Li, Xuemin Jian, Han Zhang, Jianmin Yuan, Jiajun Yin, Jianhua Chen, Yongyong Shi

**Affiliations:** 1 Bio-X Institutes, Key Laboratory for the Genetics of Developmental and Neuropsychiatric Disorders (Ministry of Education), Collaborative Innovation Center for Brain Science, Shanghai Jiao Tong University, Shanghai, 200030 People’s Republic of China; 2 Brain Science Basic Laboratory, The Affiliated Wuxi Mental Health Center With Nanjing Medical University, Wuxi, 214151 Jiangsu Province, People’s Republic of China; 3 Shanghai Clinical Research Center for Mental Health, Shanghai Key Laboratory of Psychotic Disorders, Shanghai Mental Health Center, Shanghai Jiao Tong University School of Medicine, Shanghai, 200030 People’s Republic of China; 4 Department of Psychological Medicine, Institute of Psychiatry, Psychology & Neuroscience, King’s College London, London, UK

**Keywords:** *BNIP3L* gene, Schizophrenia, Targeted next-generation sequencing, Case–control study

## Abstract

**Background:**

Schizophrenia is a chronic and severe mental disorder, and it has been predicted to be highly polygenic. Common SNPs located in or near *BNIP3L* were found to be genome-wide significantly associated with schizophrenia in recent genome-wide association studies. The purpose of our study is to investigate potential causal variants in *BNIP3L* gene.

**Results:**

We performed targeted sequencing for all exons and un-translated regions of *BNIP3L* gene among 1806 patients with schizophrenia and 998 healthy controls of Han Chinese origin. Three rare nonsynonymous mutations, *BNIP3L* (NM_004331): c.52A>G, c.167G>A and c.313A>T, were identified in schizophrenia cases, and two of them were newly reported. The frequencies of these rare nonsynonymous mutations were significantly different between schizophrenia cases and healthy controls. For the common variants, rs147389989 achieved significance in both allelic and genotypic distributions with schizophrenia. Rs1042992 and rs17310286 were significantly associated with schizophrenia in meta-analyses using PGC, CLOZUK, and our new datasets in this study.

**Conclusions:**

Our findings provided further evidence that *BNIP3L* gene is a susceptibility gene of schizophrenia and revealed functional and potential causal mutations in *BNIP3L*. However, more functional validations are suggested to better understand the role of *BNIP3L* in the etiology of schizophrenia.

## Background

Schizophrenia is a severe mental disorder that affects about 1% of the world population. The core features of schizophrenia are positive symptoms including delusions and hallucinations, negative symptoms (especially impaired motivation, reduction in spontaneous speech, and social withdrawal) and cognitive impairment [1, 2]. The first episode of schizophrenia usually occurs in late adolescence or early adulthood [3, 4]. Around 20% of schizophrenia patients have chronic symptoms and disability, and over 50% have intermittent but long-term psychiatric problems [5]. The etiology and pathogenesis of schizophrenia are not very clear, but genetic and environmental synergistic pathogeneses are generally accepted. The estimated heritability of schizophrenia is about 70–85% [6].

Schizophrenia is highly polygenic, as predicted by several genetic epidemiological researches many years ago [7]. Previous genome-wide association studies (GWASs) have revealed more than 100 distinct genetic loci are genome-wide associated with schizophrenia, which have en masse effects [1, 8, 9]. In 2017, Li et al. reported 30 new susceptibility loci of schizophrenia including rs73219805 locus near *BNIP3L* gene and predicting that the *BNIP3L* gene may be a susceptibility gene of schizophrenia [10]. Recent research about comparative genetic architectures of schizophrenia also revealed that intron variant of *BNIP3L* gene, rs117325001, was significantly associated with schizophrenia in a fixed-effect meta-analysis including individuals from East Asian and European ancestries [11].

**Fig. 1:**
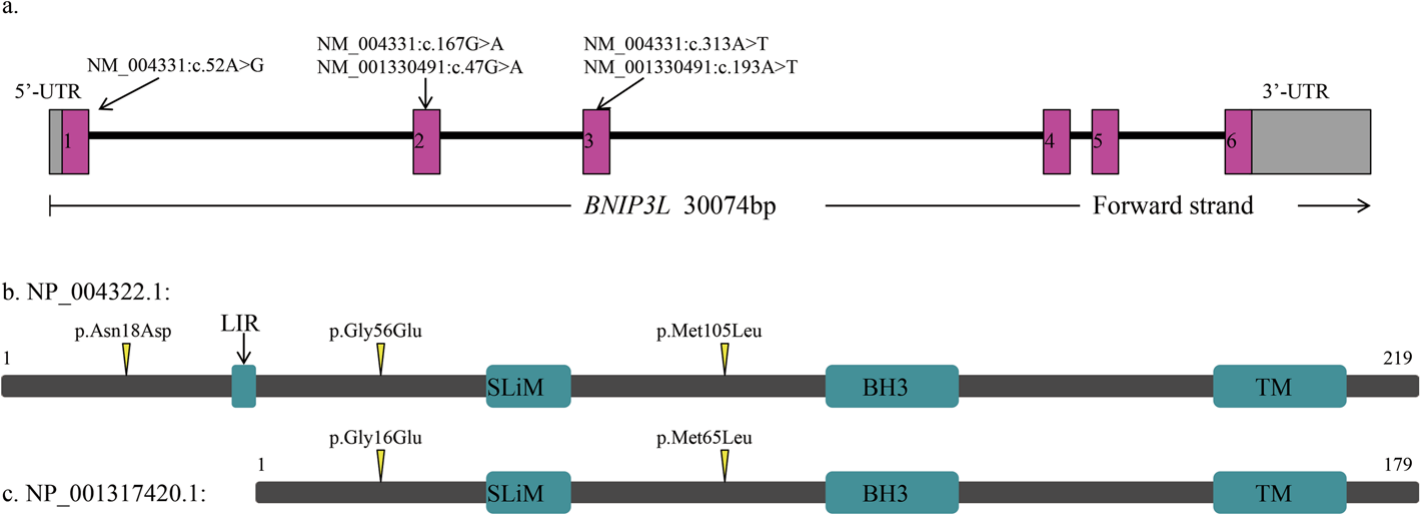
Structure of *BNIP3L* gene and NIP3L protein. **a***BNIP3L* gene structure, boxes 1~6 indicate the protein-coding exons. **b** NIP3L protein structure based on NP_0043221.1; TM is transmembrane domain. **c** NIP3L protein structure based on NP_001317420.1

The *BNIP3L* gene is located on chromosome 8p21.2 and harbors 6 exons, which encodes a 23.8-kDa protein called BCL2/adenovirus E1B 19 kDa protein-interacting protein 3-like (NIP3L/NIX) and containing a transmembrane domain and a BH3 domain in the C-terminal region. NIP3L, which belongs to the NIP3 family, has two isoforms produced by alternative splicing (Fig. 1). The protein was a single-pass, transmembrane protein, located in the nuclear envelope, endoplasmic reticulum (ER) region, and outer mitochondrial membrane (OMM) [12, 13]. The transmembrane domain of NIP3L is glycine zipper, and every two NIP3L molecules form detergent-resistant homodimers [12]. Ney stated that mitochondrial clearance in reticulocytes was affected by disruption of NIP3L’s glycine zipper, which suggested that the function realization of NIP3L requires dimerization. The majority of NIP3L protein is cytoplasmic, which contains an LC3-interacting region (LIR) motif and a novel short linear motif (SLiM) [12, 14]. LIR motif may recruit LC3 which bridges cargo with autophagy-generated membrane [15]. SLiM was inferred to interact with a hydrophobic pocket in another protein [12].

The functions of NIP3L mainly include inducing apoptosis and participating in mitochondrial quality control. On the one hand, NIP3L plays an important role in mitochondrial clearance. Previous researches have shown that a considerable proportion of erythrocytes had abnormally retained mitochondria in NIP3L-deficient mice while defective mitochondrial clearance appeared in NIX-deficient reticulocytes [16, 17]. Some studies revealed that NIP3L may increase the production of mitochondrial reactive oxygen species (ROS) and competitively bind to BCL2 (or a related protein) liberating Beclin-1 from BCL2 complexes to activate autophagy [18]. On the other hand, NIP3L also regulates cell death [12]. NIP3L causes cytochrome c release to activate apoptosis by targeting to mitochondria triggers BAX/BAK-dependent mitochondrial outer membrane permeabilization (MOMP). NIP3L activates the classic mitochondrial permeability transition (MPT) to cause necrosis by increasing ER–SR (endoplasmic reticulum–sarcoplasmic reticulum) calcium stores and releasing calcium to mitochondria [19, 20].

From all above, NIP3L affects the normal metabolism and survival of cells through quality control of mitochondria. The hypothesis of mitochondrial dysfunction is one of the important pathogenic hypotheses of schizophrenia. Bahn et al. found that glycolysis, oxidative phosphorylation, and the tricarboxylic acid cycle (TCA cycle) were significantly downregulated in the brain tissue of schizophrenia patients [21]. Kung and Roberts have reported that, in postmortem human brain tissue of off-drug schizophrenics, the numbers of mitochondria per axon terminal appeared lower than on-drug schizophrenics or controls [22]. Schizophrenia has long been considered a neurodevelopment mental disorder [23]. The immuno-inflammatory pathway is also one of the widely appreciated mechanisms while abnormal clearance of depolarized mitochondria may cause an increase of ROS and trigger a set of inflammatory responses as well as abnormal density and dysfunction of mitochondria may affect neurodevelopment [24]. Synaptic transmission is another important neurobiological process, and the destruction of it may also be one of the important pathogenic factors of schizophrenia. Mitochondria play a pivotal role in assisting synaptic transmission at synapses [25]. Firstly, the process of synaptic transmission has a high demand for adenosine triphosphates (ATPs) which are mainly provided by synaptic mitochondria [26, 27]. Secondly, mitochondria at synapses play a key role in maintaining the intra-synaptic calcium homeostasis [28] and assist the whole process of post-tetanic potentiation which may be related to memory and behavior [29, 30]. Thirdly, the synthesis, packaging, and enzymatic hydrolysis of some key neurotransmitters, such as acetylcholine, glutamate, dopamine, and serotonin, significantly rely on mitochondria [27].

All evidence shown above suggests that NIP3L may profoundly affect neuronal development through mitochondria and its biological functions probably be related to the pathogenesis of schizophrenia. In this study, we performed targeted next-generation sequencing for all exons and un-translated region (UTR) of *BNIP3L* gene in 1806 patients with schizophrenia and 998 healthy controls to identify potential pathogenic mutations of *BNIP3L* gene in schizophrenia.

## Methods

### Subjects

The sample set included 1806 unrelated schizophrenia cases (1111 men and 695 women) and 998 independent healthy controls (437 men and 561 women). The mean age is 44.49 years (standard deviation = 12.13) among schizophrenia cases and 43.15 years (standard deviation = 17.53) among healthy controls (Table 1).

**Table 1:** Characteristics of the study sample set

	*n*			Age, years	
	Men	Women	Total	Mean	s.d.
Patients with SCZ	1111	695	1806	44.49	12.13
Healthy controls	437	561	998	43.15	17.53

*SCZ* schizophrenia, *s.d.* standard deviation

The study was approved by the ethical committee. All participants were recruited from Han Chinese population and signed informed consent. All patients were diagnosed by two independent psychiatrists from the Affiliated Wuxi Mental Health Center with Nanjing Medical University. Diagnoses were made strictly according to the DSM-IV criteria based on SCID-I (the Structured Clinical Interview for DSM-IV Axis I Disorders). Patients were excluded if they had suffered mood disorder, neurological illness, mental retardation, psychotic disorder, and history of substance use due to general medical conditions. During the physical examination, healthy controls were collected.

### DNA extraction, target capture, and next-generation sequencing

LifeFeng Genomic DNA Purification Kits (Lifefeng Biotech Co., Ltd., Shanghai, China) were used to extract genomic DNA from peripheral blood samples. DNA concentration and quality were examined by NanoDrop2000 (Thermo Scientific, USA). Twenty-three pairs of primers divided into two pools were designed covering all exons, UTRs, and exon–intron boundary of *BNIP3L* gene. The sequences of primers and their targeted regions are shown in Table S1. Two-staged PCR process was performed for library construction. All the PCR reagents and protocol were supported by Shanghai DYnastyGene Company. High Sensitivity DNA kits and 2100 Bioanalyzer (Agilent Technologies, USA) were used to determine the size distribution of DNA library fragments. The Illumina HiSeq X Ten System (Illumina, USA) was used to sequence the final DNA libraries as PE 150 bp reads.

### Variant identification and validation

Genome Analysis Toolkit (GATK) Best Practices [31], the pipeline for germline short variant discovery, was run on every data set independently. Burrows–Wheeler Aligner (BWA) [32] was used to align raw reads to the human reference genome (GRCh38), GATK haplotypercaller was used to call variants (single-nucleotide polymorphisms, SNPs; short insertions and deletions, InDels), and Annovar [33] was used to annotate the variants. Multi-species alignments were performed using Clustal Omega online software [34]. Sanger sequencing was performed to verify the rare nonsynonymous variants.

### Case–control study

Single locus association tests, Hardy–Weinberg equilibrium (HWE), and pairwise linkage disequilibrium (LD) analysis were performed on the SHEsisPlus online software platform [35–37]. For the common variants, pairwise LD analysis was performed and adjacent loci with D′ > 0.90 were classified in the same block. The chi-square test and Fisher’s exact test were used to infer whether polymorphisms of the alleles and genotypes were associated with schizophrenia. Fisher’s exact test would be used if the theoretical frequency less than five occurred in any group. All tests were two-tailed, and statistical significance was set at *P* < 0.05. The false discovery rate (FDR-BH) was used to calibrate the *P* values.

### Meta-analyses

CLOZUK and Independent PGC2 datasets were used for the meta-analysis with our data. We performed meta-analyses by RevMan5.3. Heterogeneity analysis of the odds ratios was tested by Cochran’s Q test and *I*^2^. No significant heterogeneity existed, and fixed effects model was used when *P* > 0.05 and *I*^2^ < 50%; otherwise, random effects model would be selected [38].

## Results

### Variants identification

We performed Sanger sequencing validation for ten samples containing missense mutations detected by next-generation sequencing. Among them, the three mutations of eight samples were verified to be true positives, and their next-generation sequencing depths are all deeper than 20 ×. The other 2 samples were verified to be false positives, and their depths were all lower than 10 ×. Therefore, only the variants with a depth more than 10 × were included in further analysis.

A total of 114 high-quality variants were identified in case and control groups including six variants in coding exons, 25 intron variants, one upstream variant, and 82 UTR variants (Table S2). Three databases, the 1000 Genomes Project [39], the Exome Aggregation Consortium [40], and NHLBI Exome Sequencing Project [41] were used to filter rare mutations. Only loci with minor allele frequency (MAF) less than 0.01 in each database were selected, and in total, 105 rare mutations including 69 novel variants were detected. The other nine variants are common SNPs, and their information is shown in Table S3.

### Analyses of rare variants in coding regions

**Fig. 2:**
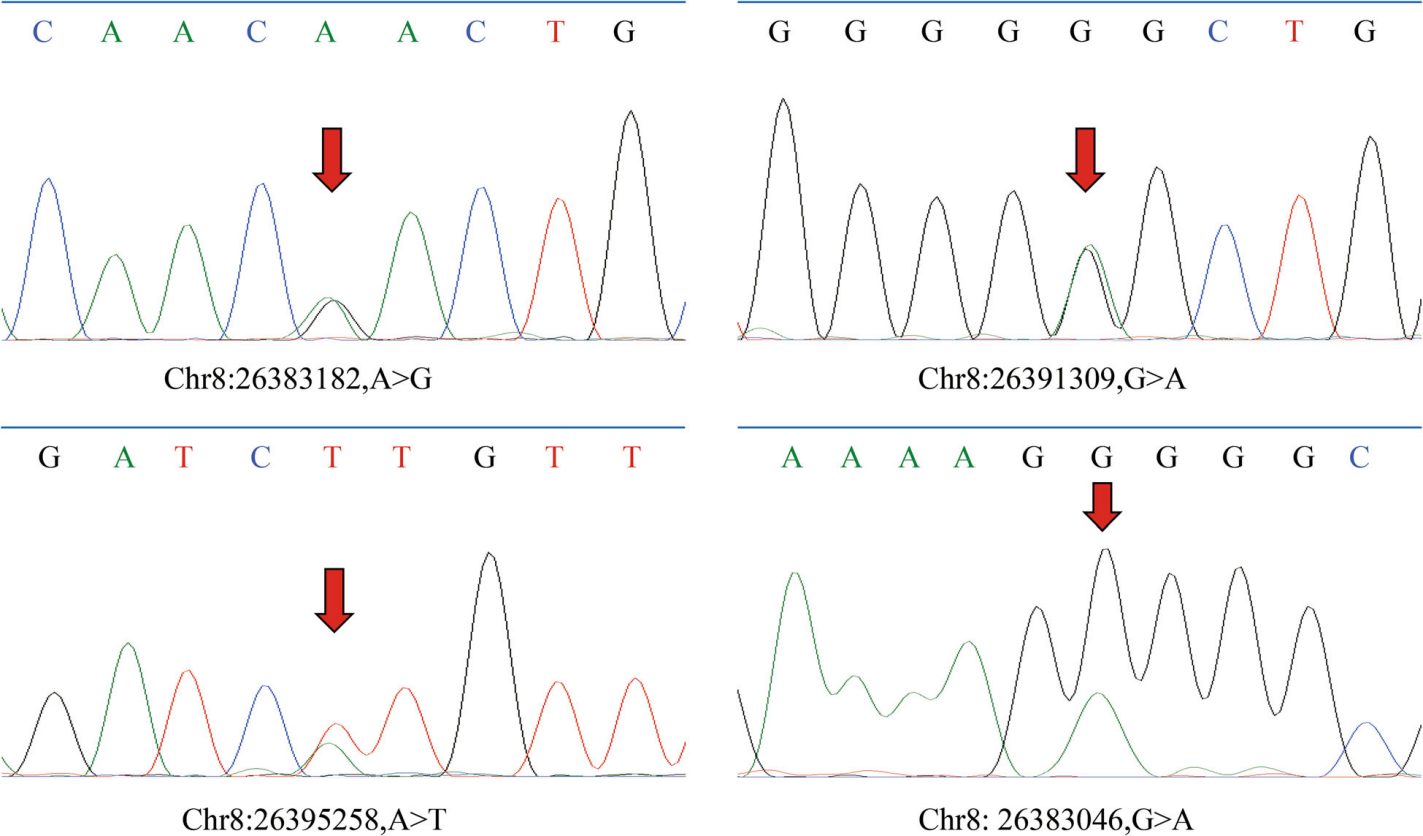
The results of Sanger sequence verifying the rare nonsynonymous variants. Arrows indicate the mutation sites

In total, six variants in coding exons including two synonymous mutations, one nonframeshift deletion, and three nonsynonymous mutations (Fig. 1) were identified. They are all heterozygous and rare mutations. Among them, only one synonymous mutation was detected from one sample of the control group. Other mutations were all from the case group and were detected in 12 independent patients, of which eight patients were with the nonsynonymous mutations. We performed Sanger sequencing to verify all the nonsynonymous variants, *BNIP3L* (NM_004331): c.52A>G, c.167G>A and c.313A>T. The results are shown in Fig. 2. All variants were verified to be heterozygous mutations. In addition, c.167G>A and c.313A>T were newly reported.

PolyPhen-2 [42], MutationTaster [43], SIFT [44], and LRT [45] in Annovar were used to predict the pathogenicity of the variants (Table 2). All the three variants are predicted to be “disease causing” by MutationTaster. By SIFT, c.52A>G and c.167G>A were predicted to be deleterious and c.313A>T to be tolerated. By LRT, c.167G>A and c.313A>T were predicted to be deleterious as well as c.52A>G to be neutral. Moreover, by Polyphen-2, c.52A>G was predicted to be possibly damaging, c.167G>A to be probably damaging, and c.313A>T to be benign respectively. We also access the CADD (Combined Annotation Dependent Depletion) score [46] of the three variants, and their scores are all greater than 20, which means that the variants are among the top 1% of deleterious variants in the human genome.

**Table 2:** Detailed information of rare mutations detected in this study

Variants	Position	Variants status	SIFT	Polyphen-2	MutationTaster	LRT	CADD score	Novel or not	Individuals	Gender	Group
NM_004331: c.52A>G/p.Asn18Asp	chr8:26383182	Heterozygous	D	P	D	N	23.6	rs549425213	55S0002	Male	SCZ
									55S0703		
									DI226		
									55S0439	Female	
									DI182		
NM_004331: c.167G>A/p.Gly56Glu, NM_001330491: c.47G>A/p.Gly16Glu	chr8:26391309	Heterozygous	D	D	D	D	31	novel	YZ005	Male	SCZ
NM_004331: c.313A>T/p.Met105Leu, NM_001330491: c.193A>T/p.Met65Leu	chr8:26395258	Heterozygous	T	B	D	D	22.1	novel	SYS0036	Female	SCZ
									DI010		

*D* deleterious for SIFT and LRT, probably damaging for Polyphen-2, disease_causing for MutationTaster; *T*, tolerated; *P*, possibly damaging; *B*, benign; *N*, neutral; *SCZ*, schizophrenia; *CADD*, Combined Annotation Dependent Depletion

**Fig. 3:**
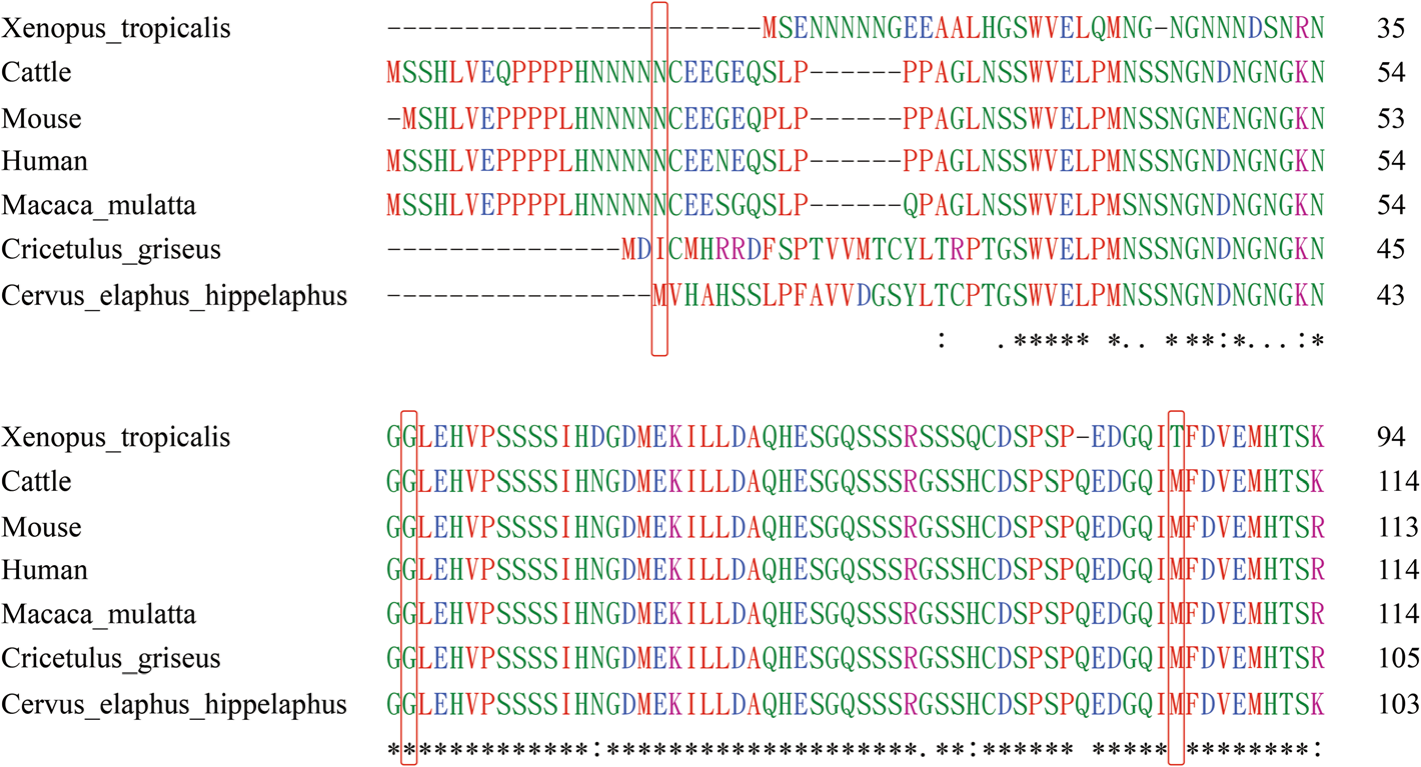
Multiple alignments of NIP3L protein sequences of various species. The sites of nonsynonymous variants were box out

The amino acid variants for the three rare nonsynonymous mutations are *BNIP3L* (NP_004322): p.Asn18Asp, p.Gly56Glu, and p.Met105Leu. Multiple alignments of NIP3L protein sequences from several available species showed that p.Gly56Glu is located in a highly conserved region evolutionally while the other two loci, p.Asn18Asp and p.Met105Leu, are not so that conserved (Fig. 3).

### Association analysis of all variants

Case–control study was performed for all the variants except for four single-nucleotide variants (SNVs) including one rare and three common variants, because these four SNVs did not reach Hardy–Weinberg equilibrium in the healthy controls. No significant association was observed between rare variants and schizophrenia in our sample set. Although the rare variant, rs549425213 (*P*_allele_ = 0.096 before correction), and our novel reported variant in 5′-UTR, NM_004331:c.-85G>A (*P*_allele_ = 0.068 before correction), are not significantly associated with schizophrenia, the variants are only detected in cases and their *p* values are very close to 0.05. We also performed gene-based association study for nonsynonymous mutations. In total, eight patients with schizophrenia carried nonsynonymous mutations which were not detected in the control group. The chi-square test was performed, and the accumulated number of nonsynonymous mutation carriers in the case group and control group was significantly different (chi^2^ = 4.433, *P* = 0.035).

The results for common variants are shown in Table 3. Rs147389989 achieved both allelic and genotypic significance after FDR-BH correction (*P*_allele_ = 0.007, *P*_genotype_ = 0.017, odds ratio (OR) for “G” [95% confidence interval (CI)] = 2.383[1.536~3.698]), and the allele “G” was suggested to be a risk factor for schizophrenia. Before FDR-BH correction, rs1055476 (*P*_allele_ = 0.004, *P*_genotype_ = 0.004, OR for “-” [95%CI] = 3.245[1.362~7.728]) and rs17310286 (*P*_allele_ = 0.012, *P*_genotype_ = 0.028, OR for “T” [95%CI] = 1.154 [1.031~1.291]) were significantly associated with schizophrenia both in allelic and genotypic distributions, but there was no significance after correction.

**Table 3:** Association results of 6 common variants

SNP ID	Group	Allele frequency		Allelic *P*	Corrected *P*^a^	O*R*	95% CI	Genotype frequency			Genotypic *P*	Corrected *P*^a^
rs1055476		G	C					C/C	C/G			
chr8:26383121	SCZ	35 (0.009)	3577 (0.990)	0.004**	0.179	3.245	[1.362~7.728]	1771 (0.980)	35 (0.019)		0.004**	0.174
	Control	6 (0.003)	1990 (0.996)					992 (0.993)	6 (0.006)			
rs147389989		-	TCTT					TCTT/TCTT	TCTT/-	-/-		
chr8:26411153	SCZ	106 (0.029)	3506 (0.970)	6.52e−05**	0.007**	2.383	[1.536~3.698]	1701 (0.941)	104 (0.057)	1 (5.54e−04)	3.22e-04**	0.017*
	Control	25 (0.012)	1971 (0.987)					973 (0.974)	25 (0.025)			
rs17310286		T	C					C/C	T/C	T/T		
chr8:26411516	SCZ	1462 (0.404)	2150 (0.595)	0.012*	0.266	1.154	[1.031~1.291]	636 (0.352)	878 (0.486)	292 (0.161)	0.028*	0.452
	Control	740 (0.370)	1256 (0.629)					385 (0.385)	486 (0.486)	127 (0.127)		
rs17055200		A	G					G/G	G/A	A/A		
chr8:26411573	SCZ	134 (0.037)	3478 (0.962)	0.576		0.922	[0.695~1.223]	1673 (0.926)	132 (0.073)	1 (5.54e-04)	0.604	
	Control	80 (0.040)	1916 (0.959)					918 (0.919)	80 (0.080)			
rs1042992		T	C					C/C	T/C	T/T		
chr8:26411675	SCZ	1435 (0.397)	2177 (0.602)	0.581		1.031	[0.922~1.154]	662 (0.366)	853 (0.472)	291 (0.161)	0.385	
	Control	778 (0.389)	1218 (0.610)					363 (0.363)	492 (0.492)	143 (0.143)		
rs10503786		T	C					C/C	C/T	T/T		
chr8:26412420	SCZ	656 (0.181)	2956 (0.818)	0.833		1.015	[0.880~1.170]	1215 (0.672)	526 (0.291)	65 (0.035)	0.674	
	Control	358 (0.179)	1638 (0.820)					670 (0.671)	298 (0.298)	30 (0.030)		

*SCZ* schizophrenia, *SNP* single-nucleotide polymorphism, *OR* odds ratio, *CI* confidence interval

*P values < 0.05

**P values < 0.01

^a^FDR correction

**Fig. 4:**
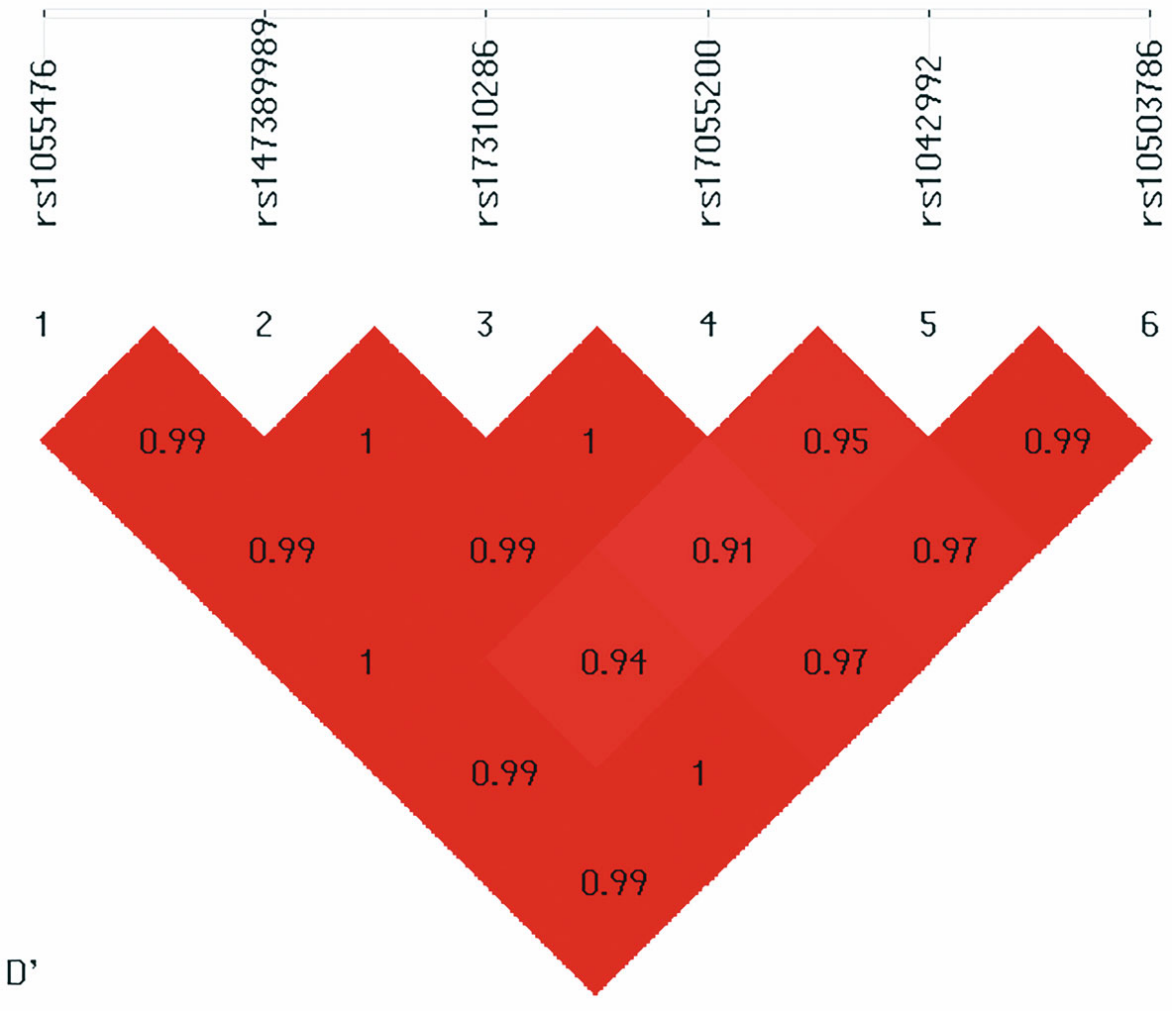
Pairwise linkage disequilibrium plot for the common variants in *BNIP3L* gene. The pairwise D′ values are presented in the matrices; deep red implicates relatively strong linkage disequilibrium and vice versa

Pairwise LD analysis indicated that all the six common variants formed one haplotype block (Fig. 4). With frequency > 0.03, four haplotypes, C-TCTT-C-G-C-C/C-TCTT-T-G-T-C/C-TCTT-C-G-C-T/C-TCTT-C-A-C-C, were identified. Haplotype “C-TCTT-T-G-T-C” was implied to be a significant risk factor for schizophrenia (*P* = 5.95 × 10^−4^ after FDR correction, OR [95%CI] = 1.225 [1.093~1.373]). The global Pearson’s *p* is 0.048 (Table 4).

**Table 4:** Results of haplotype analysis

Haplotype^a^	Case frequency	Control frequency	Chi^2^	*P*	Corrected *P*^b^	OR [95% CI]	Global Chi^2^	Global *P*
C-TCTT-C-G-C-C	1209 (0.334)	707 (0.354)	2.171	0.140	0.162	0.917 [0.817 ~ 1.028]	7.904	0.048*
C-TCTT-T-G-T-C	1408 (0.389)	684 (0.342)	12.207	4.76e-04**	5.95e-04**	1.225 [1.093 ~ 1.373]		
C-TCTT-C-G-C-T	643 (0.178)	349 (0.174)	0.088	0.765	0.820	1.022 [0.885 ~ 1.179]		
C-TCTT-C-A-C-C	131 (0.036)	72 (0.036)	0.001	0.970	0.970	1.005 [0.750 ~ 1.347]		

*OR* odds ratio, *CI* confidence interval

*P values < 0.05

**P values < 0.01

^a^Haplotypes with frequency < 0.03 were ignored

^b^FDR correction

### The results of meta-analyses

**Fig. 5:**
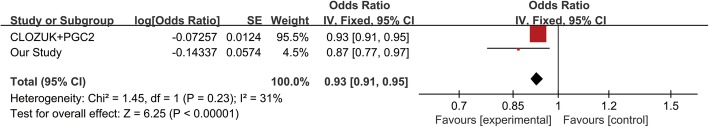
Forest plot of meta-analysis result for rs17310286

**Fig. 6:**
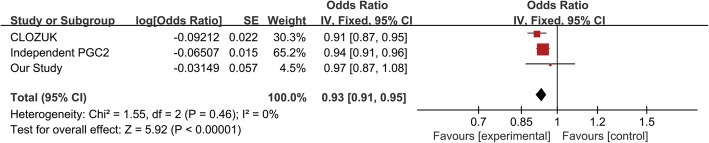
Forest plot of meta-analysis result for rs1042992

From the results of previous GWASs [8, 9] and SZDB2.0 database [47, 48], rs17310286 and rs1042992 were genotyped before. Therefore, the results of previous GWAS and our data were used for meta-analysis (Table 5). CLOZUK and independent PGC datasets were used for meta-analyses with our results. No heterogeneity was detected in the combined sample sets (rs17310286: *I*^2^ = 31%, chi^2^ = 1.45, *P* = 0.23; rs1042992: *I*^2^ = 0%, chi^2^ = 1.55, *P* = 0.46), so fixed effects model was chosen. Although these two loci were not significantly associated with schizophrenia in our sample set, significant associations were observed after meta-analyses (rs17310286: pooled OR [95%CI] = 0.93[0.91–0.95], *Z* = 6.25, and *P* < 1.00 × 10^−4^; rs1042992: pooled OR [95%CI] = 0.93[0.91–0.95], *Z* = 5.92, and *P* < 1.00 × 10^−4^) as shown in forest plot of Figs. 5 and 6. The alternate allele “T” for the both two loci were suggested to be risk factors for schizophrenia which was consistent with the results of previous GWAS research [9].

**Table 5:** Results of meta-analysis

Variants	Allele	Data set	OR	SE	*P* value	Model	Heterogeneity *I*^2^	Heterogeneity *P* value	Total OR [95%CI]	Overall Effect (*Z*)	Total *P* value
rs17310286	C/T	CLOZUK+PGC2	0.930	0.012	5.43e−09**	Fixed	31%	0.23	0.93 [0.91~0.95]	6.25	*P* < 1.00e−05**
chr8:26411516		Our Study	0.867	0.057	0.012*						
rs1042992	C/T	Independent PGC2	0.937	0.015	1.94e−05**	Fixed	0%	0.46	0.93 [0.91~0.95]	5.92	*P* < 1.00e−05**
chr8:26411675		CLOZUK	0.912	0.022	2.79e−05**						
		Our Study	0.970	0.057	0.581						

Our study: Han Chinese population

*OR* odds ratio, *CI* confidence interval

*P values < 0.05

**P values < 0.01

### Discussion

In our results, we found three rare nonsynonymous mutations in the *BNIP3L* gene, c.52A>G, c.167G>A and c.313A>T, carried by eight patients with schizophrenia. Among them, c.167G>A and c.313A>T were newly reported and all of them were predicted to be disease-causing variants by MutationTaster. All three mutations are located in the extra-membrane region of the NIP3L protein, suggesting that the variants may affect the interaction of NIP3L with other proteins and may play a key role in the pathogenesis of schizophrenia. Our novel reported variant in 5′-UTR, NM_004331:c.-85G>A, is carried by six patients with schizophrenia which is not detected in the control group. We also performed the Sanger sequencing to verify *BNIP3L* (NM_004331): c.-85G>A, and the results are shown in Fig. 2. In the case DI226, heterozygous c.-85G>A and c.52A>G (nonsynonymous mutations) were detected as bi-allelic mutations for schizophrenia. The CADD score of c.-85G>A is 20.6, and it is predicted to be “disease causing” by MutationTaster. As predicted by MutationTaster, c.-85G>A might cause splice site changes and affect protein features. The results of the gene-based association analysis showed that the number of nonsynonymous mutations carried by schizophrenia patients was significantly more than healthy controls, which further confirmed that the *BNIP3L* gene is a susceptibility gene of schizophrenia.

We found rs147389989 in the *BNIP3L* gene was significantly associated with schizophrenia, and we searched for this locus in the SZDB database [47]. We found that rs147389989 has been genotyped in schizophrenia cases and healthy controls in CLOZUK sample sets [9]. But they did not observe any significant association between rs147389989 and schizophrenia. In our study, all participants were recruited from Han Chinese population while CLOZUK samples were from European, and this may cause the inconsistency between our results and previous study. The recent genome-wide association study [9] of schizophrenia identified 145 associated loci through meta-analysis, and rs1042992 (*P* = 3.67 × 10^−9^, OR for allele “C” = 0.93, standard error (SE) = 0.012) is one of the 50 novel associated loci. Moreover, rs17310286, which highly linked with rs1042992, was also significantly associated with schizophrenia (*P* = 5.43 × 10^−9^, OR for allele “C” = 0.93, SE = 0.012). But in our results, the significant association between rs1042992 and schizophrenia was not observed. Although rs17310286 was significantly associated with schizophrenia both in allelic and genotypic distributions, there was no significance after correction. So we performed meta-analyses using the results of the GWAS and our study. No heterogeneity was detected in the combined sample sets. These two loci were both significantly associated with schizophrenia in meta-analyses and had the same direction of effect with the previous GWAS. Our negative results of these two loci may be caused by the limited size of our sample set. From the results of haplotype analysis of our data, we found that the haplotype, C-TCTT-T-G-T-C, was a significant risk factor for schizophrenia and the two loci, rs1042992 and rs17310286, were both the risk allele “T” for the haplotype which was consistent with the results of meta-analyses. The results of meta-analyses and the haplotype analysis further confirmed that rs1042992 and rs17310286 located in 3′-UTR of *BNIP3L* gene were significantly associated with schizophrenia and provided more evidence for *BNIP3L* gene as a candidate gene for schizophrenia.

The three common variants significantly associated with schizophrenia in our study are located in the 3′-UTR of the *BNIP3L* gene. It is suggested that the 3′-UTR of the *BNIP3L* gene may play a crucial role in schizophrenia. 3′-UTR is a pivotal region which regulates gene expression, protein–protein interactions, and mRNA localization by binding with micro-RNAs (miRNAs) or RNA-binding proteins (RBPs) [49, 50]. Several studies have reported that some SNPs affect the risk of schizophrenia because their alter alleles can change the binding stability of some miRNAs with the 3′-UTR of the gene [51–53]. Dysregulation of some miRNAs is associated with central nervous system diseases including schizophrenia [54].

In our study, for rs1042992 and rs17310286, we used SNPinfo Web Server [55] to predict their possible functions. We found that the allele “T” of rs1042992 would create the binding of hsa-miR-495 and hsa-miR-590-3p with the 3′-UTR. Interestingly, previous research has shown that hsa-miR-495 was one of the human miRNAs that were significantly differentially expressed (*P* < 0.05) between autistic and nonautistic individuals [56]. Hsa-miR-495 was also identified to involve in mechanisms to enhance neuroprotection [57] and selectively affect addiction-related behaviors [58]. Moreover, hsa-miR-590-3p was proven to involve in mitochondrial dysfunction and oxidative stress and play a key role in the pathogenesis of Parkinson’s disease [59]. All of the above suggested that the polymorphism of rs1042992 and rs17310286 might regulate the expression of NIP3L by changing some miRNAs binding and affect the risk of schizophrenia.

Our study identified several variants in *BNIP3L* gene that may affect the expression and function of NIX, while the role of NIX in mitochondrial clearance and apoptosis has been verified which is important for neuron protection in brain. Yuan et al. revealed the involvement of NIX in cerebral ischemia–reperfusion (I-R)-induced mitophagy which could attenuate brain injury after cerebral ischemia. Their research found that *Bnip3l* knockout (*bnip3l-/-*) impaired mitophagy and aggravated cerebral I-R injury in mice, which can be rescued by NIX overexpression [60]. Ma et al. reported that in a rat traumatic brain injury model, NIX expression colocalized with neuronal cells in cortical areas. Moreover, autophagy increased and neuronal apoptosis decreased after inducing upregulation of NIX. They suggested that NIX probably plays a neuroprotective role in rat traumatic brain injury through autophagy and apoptosis pathways [61]. Therefore, *BNIP3L* may protect neurons through mitochondrial quality control and may be related to the pathogenesis of some mental diseases.

Our results provided additional evidence that *BNIP3L* was a susceptibility gene of schizophrenia. However, there are two limitations of our study. One is the limited sample size which may be the reason why no significant association between single rare nonsynonymous variants and schizophrenia was observed. And the other is the lack of functional verification. Targeted sequencing for *BNIP3L* gene among more patients with schizophrenia and more functional validations are considered to be necessary for further understanding the etiology correlated with *BNIP3L* in schizophrenia.

## Conclusions

Our research presented a comprehensive mutation spectrum of *BNIP3L* gene in schizophrenia. In *BNIP3L*, we found that the number of total rare nonsynonymous mutation carriers in schizophrenia cases and healthy controls were significantly different. For the common variants, we found rs147389989 was significantly associated with schizophrenia in Han Chinese population. By meta-analyses, we also confirmed rs1042992 and rs17310286 were both significantly associated with schizophrenia. All the results further confirmed that *BNIP3L* gene is a susceptibility gene of schizophrenia, but further functional validations are needed for understanding the role of *BNIP3L* in the pathogenesis of schizophrenia.

## Supplementary information

**Additional file 1: Table S1.** The sequences of primers and their targeted regions. **Table S2.** A total of 114 high-quality variants were identified in case and control groups. **Table S3.** Detailed information of the 9 common variants in the *BNIP3L* Gene.

## Data Availability

All data generated or analyzed during this study are included in this published article and its supplementary information files.

## Abbreviations

SNP: Single nucleotide polymorphism
GWASs: Genome-wide association studies
ER: Endoplasmic reticulum
OMM: Outer mitochondrial membrane
LIR: LC3-interacting region
SLiM: Short linear motif
ROS: Reactive oxygen species
MOMP: Mitochondrial outer membrane permeabilization
MPT: Mitochondrial permeability transition
SR: Sarcoplasmic reticulum
TCA cycle: The tricarboxylic acid cycle
ATPs: Adenosine triphosphates
UTR: Un-translated region
GATK: Genome Analysis Toolkit
BWA: Burrows–Wheeler Aligner
HWE: Hardy–Weinberg equilibrium
LD: Linkage disequilibrium
FDR-BH: False discovery rate
MAF: Minor allele frequency
CADD: Combined Annotation Dependent Depletion
SNVs: Single nucleotide variants
OR: Odds ratio
CI: Confidence interval
SE: Standard error
miRNAs: Micro-RNAs
RBPs: RNA-binding proteins
I-R: Ischemia–reperfusion
